# Supplementary material for: Kolmogorov Complexity of Coronary Sinus Atrial Electrograms Before Ablation Predicts Termination of Atrial Fibrillation After Pulmonary Vein Isolation
Source: Entropy (Basel). 2019 Oct 4;21(10):970. doi: 10.3390/e21100970 (PMC7514301; doi:10.3390/e21100970)
Supplement: Supplementary file 1 [file entropy-21-00970-s001.zip › suppMat/TableS1.docx]

**Table S1** Parameters used for the calculation of complexity methods.

| **method** | **parameter** | **Value/way of calculation** |
| --- | --- | --- |
| Shannon entropy  (ShEn) | bin size | 0.25 * SD of data vector |
| dominant frequency  (DF) | Butterworth filtering bandpass | [40,250] Hz |
|  | Butterworth filtering bandpass | order = 3 |
|  | minimal DF | 3 |
|  | maximal DF | 12 |
| NaVX CFAE | refractory period | 40 ms |
|  | peak-to-peak sensitivity | 0.15 mV |
|  | duration (for farfield removal) | 10 ms |
| CFAE | minimal ICL threshold | 0.05 mV |
|  | maximal ICL threshold | 0.15 mV |
|  | minimal ICL duration | 50 ms |
|  | maximal ICL duration | 110 ms |
|  | interval length for CEA | 50 ms |
| sample entropy | matching tolerance *r* | 0.1 |
|  | maximal template length *m* | 5 |
|  | length of time series *N* | equal to analysed window length |
