# Supplementary material for: Kolmogorov Complexity of Coronary Sinus Atrial Electrograms Before Ablation Predicts Termination of Atrial Fibrillation After Pulmonary Vein Isolation
Source: Entropy (Basel). 2019 Oct 4;21(10):970. doi: 10.3390/e21100970 (PMC7514301; doi:10.3390/e21100970)
Supplement: Supplementary file 1 [file entropy-21-00970-s001.zip › suppMat/TableS2.docx]

**Table S2**  Results obtained for prior to termination recordings. Calculations were performed by analogy to the calculations for baseline. In the table p-values and AUC ROC values calculated for the performance of all the methods for discriminating patients with AF terminated after only PVI and for patients for whom additional ablation stages were performed are presented.

|  | **p-value** | | | | | | **ROC** | | | |
| --- | --- | --- | --- | --- | --- | --- | --- | --- | --- | --- |
| **method** | | **2s** | **5s** | **10s** | **30s** | **2s** | | **5s** | **10s** | **30s** |
| **LZC** | | 0.34 | 0.39 | 0.23 | 0.22 | 0.58 | | 0.58 | 0.63 | 0.65 |
| **BDM** | | 0.35 | 0.31 | 0.17 | 0.17 | 0.58 | | 0.60 | 0.66 | 0.66 |
| **AFCL** | | 0.70 | 0.94 | 0.70 | 0.99 | 0.44 | | 0.51 | 0.54 | 0.52 |
| **DF** | | 0.31 | 0.13 | 0.57 | 0.75 | 0.38 | | 0.65 | 0.44 | 0.43 |
| **DF RI** | | 0.62 | 0.22 | 0.40 | 0.52 | 0.56 | | 0.34 | 0.41 | 0.53 |
| **DF OI** | | 0.77 | 0.14 | 0.39 | 0.70 | 0.47 | | 0.36 | 0.65 | 0.43 |
| **ShEn** | | 0.75 | 0.54 | 0.36 | 0.25 | 0.58 | | 0.58 | 0.62 | 0.61 |
| **SampEn** | | 0.20 | 0.24 | 0.32 | 0.34 | 0.33 | | 0.37 | 0.38 | 0.41 |
| **CEA** | | 0.41 | 0.63 | 0.85 | 0.72 | 0.43 | | 0.45 | 0.49 | 0.49 |
| **ICL** | | 0.99 | 0.88 | 0.71 | 0.54 | 0.52 | | 0.57 | 0.54 | 0.43 |
| **ACI** | | 0.84 | 0.88 | 0.09 | 0.60 | 0.54 | | 0.57 | 0.69 | 0.52 |
| **SCI** | | 0.74 | 0.95 | 0.11 | 0.24 | 0.54 | | 0.59 | 0.63 | 0.38 |
| **NAVX** | | 0.65 | 0.85 | 0.18 | 0.74 | 0.58 | | 0.61 | 0.75 | 0.72 |
| **WMSI** | | 0.11 | 0.62 | 0.94 | 0.85 | 0.67 | | 0.55 | 0.48 | 0.52 |
